# Recent Trends in Therapeutic Approaches for Diabetes Management: A Comprehensive Update

**DOI:** 10.1155/2015/340838

**Published:** 2015-07-27

**Authors:** Pragya Tiwari

**Affiliations:** ^1^Department of Metabolic and Structural Biology, Central Institute of Medicinal and Aromatic Plants (CSIR-CIMAP), P.O. Box CIMAP, Lucknow, Uttar Pradesh 226015, India; ^2^Molecular Biology and Biotechnology Division, ICAR-National Bureau of Fish Genetic Resources, Canal Ring Road, P.O. Dilkusha, Lucknow, Uttar Pradesh 226002, India

## Abstract

Diabetes highlights a growing epidemic imposing serious social economic crisis to the countries around the globe. Despite scientific breakthroughs, better healthcare facilities, and improved literacy rate, the disease continues to burden several sections, especially middle and low income countries. The present trends indicate the rise in premature death, posing a major threat to global development. Scientific and technological advances have witnessed the development of newer generation of drugs like sulphonylureas, biguanides, alpha glucosidase inhibitors, and thiazolidinediones with significant efficacy in reducing hyperglycemia. Recent approaches in drug discovery have contributed to the development of new class of therapeutics like Incretin mimetics, Amylin analogues, GIP analogs, Peroxisome proliferator activated receptors, and dipeptidyl peptidase-4 inhibitor as targets for potential drugs in diabetes treatment. Subsequently, the identification and clinical investigation of bioactive substances from plants have revolutionized the research on drug discovery and lead identification for diabetes management. With a focus on the emerging trends, the review article explores the current statistical prevalence of the disease, discussing the benefits and limitations of the commercially available drugs. Additionally, the critical areas in clinical diabetology are discussed, with respect to prospects of statins, nanotechnology, and stem cell technology as next generation therapeutics and why the herbal formulations are consistently popular choice for diabetes medication and management.

## 1. Introduction

**Figure 1:**
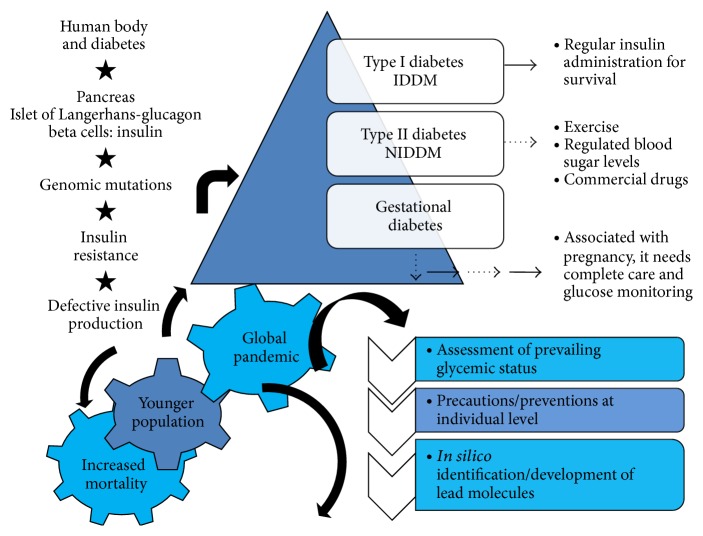
A schematic representation of general occurrence and approaches in diabetes management.

Diabetes is a major killer worldwide and its unprecedented rise poses a serious threat to mankind. According to recent estimation, 387 million people worldwide are affected from the disease with a prevalence rate of 8.3% and 46.3% still remains undiagnosed [1]. Furthermore, maximum percentage of 387 million people lives in low and middle income countries and comprise of 40–59 age group in the population. Population survey by the Indian Council of Medical Research [2] suggested that China leads the survey with an estimation of 98.4 million cases and India coming next with 65.1 million diabetes patients [3]. It has been seen that certain features in Asian Indians make them more prone to diabetes and coronary artery disease [4, 5] which include increased insulin resistance [6] and greater abdominal adiposity as reported [7].  Figure 1 depicts a schematic representation of general occurrence and approaches in diabetes management.

The selection and application of a glucose lowering therapy are dependent on a number of considerations like the severity of hyperglycemia, hepatic and renal associated functions, risks of hypoglycemia, body mass index, ability to self monitor the blood glucose level, and also the cost of the medication. The therapeutics for type 1 diabetes includes stimulation of insulin secretion through GLP analogues like Exenatide and Liraglutide [8, 9], insulin injections to compensate for *β* cell defects, dipeptidyl peptidase-4 (DPP-4) inhibition by Sitagliptin, and increased islet survival [10, 11] and islet cell regeneration through islet neogenesis associated protein (INGAP) peptide therapy aiming at islet cell regeneration among others [12].

The treatment approach for type 2 diabetes includes several conventional therapeutics, namely, sulfonylureas and repaglinide enhance insulin secretion, troglitazone increases insulin action in fat and muscle, metformin promotes insulin mechanism in liver tissue, and miglitol and acarbose enact delayed carbohydrate absorption from food intake, respectively [13]. The drugs used for the treatment of type 2 diabetes poses limitations in the sense that they have significant side effects. The other major medications strategies constitutes combinational therapy of insulin with sulfonylureas which reduced the daily requirement of insulin [14] and insulin and metformin combination therapy (approved by FDA); minimizing weight gain due to insulin therapy [15] and troglitazone-insulin in combination efficiently reduced insulin requirement and improved glycemic control [16].

*Secondary Forms of Diabetes.* Subsequently, secondary forms of the disease may arise due to defects/mutations in genome of the organism: gene mutations in MODY1-hepatocyte nuclear factor-4-alpha (HNF4*α*) in chromosome 20q12-q13.1; MODY2-glucokinase (GCK) gene mutation in chromosome 7p15-p13; MODY3-hepatocyte nuclear factor-1-alpha (HNF1A) gene mutation in chromosome 12q24.2; MODY4-insulin promoter factor-1 (IPF1) gene mutation in chromosome 13q12.1; MODY5-hepatic transcription factor-2 (TCF2) gene mutation, chromosome 17cen-q21.3; MODY6- neurogenic differentiation 1 (NEUROD1) gene mutation in chromosome 2q32; MODY7-Kruppel-like factor 11 (KLF11) gene mutation in chromosome 2p25; MODY8 (or diabetes-pancreatic exocrine dysfunction syndrome)-carboxylester lipase (CEL) gene mutation, chromosome 9q34; MODY9-paired box gene 4 (PAX4) mutation, chromosome 7q32:

- *Mutations occurring in mitochondrial genome,* referred to as mitochondrial diabetes MELAS syndrome (mitochondrial myopathy, stroke-like syndrome encephalopathy, and lactic acidosis).
- *Genetic syndromes*, Klinefelter's syndrome, Turner's syndrome, Down syndrome, Prader-Willi, Laurence-Moon-Biedl, Friedreich's ataxia, Huntington's chorea, myotonic dystrophy, and porphyria.
- *Drug/chemical induced diabetes,* Thiazides, DPH, *α*-interferon, L-asparaginase, vacor, nicotinic acid, pentamidine, steroids, levothyroxine, and diazoxide [17].

However, the disease together with its multiple complications puts forward the immediate requirement to act with a well defined strategy. The underlying platform is aimed at achievement of complete glycemic regulation, possible through assessment of present glycemic status, and analysis of the associated disorder would aim at allocating the healthcare facilities to the affected people [18]. The new generation of drugs like sulphonylureas or insulin can induce hypoglycemia as well as weight gain [19] while the biguanide like metformin can cause gastrointestinal effects such as diarrhoea and nausea and, rarely, lactic acidosis. Thiazolidinedione use is also associated with weight gain, which is an issue of concern since type 2 diabetic patients are already obese [20]. Recent generation of drugs like the incretin mimetics may produce nausea, vomiting, and diarrhoea [21]. The drugs showing potential for the cure of diabetes have been used singly and also in combination of multiple oral agents and with addition of insulin but achieving the complete glycemic control is a challenging task.

The present trends in diabetes therapeutics and management have highlighted an urgent requirement for extensive investigations aiming at identification and clinical trials of natural products and their analogues in drug discovery studies. In an attempt to address the global issue, the paper provides a comprehensive update highlighting the global scenario and statistical prevalence of diabetes. Furthermore, the emerging trends in clinical diabetology were discussed, exploring the advantages as well as the limitations of the commercially available therapeutics. The present era has witnessed the development of next generation therapeutics, statins, nanotechnology, and stem cell technology and the reasons why the natural products and analogues define a prospective field in diabetes medication and management.

## 2. Classification of Diabetes

On the basis of insulin deficiency, diabetes can be classified into the following types as follows.

### 2.1. Insulin Dependent Diabetes Mellitus (IDDM)

It is also known as juvenile onset diabetes or type 1 diabetes, which accounts for 5–10% of the patients, resulting from cellular-mediated autoimmune destruction of the pancreatic cells. The disease can affect people of all ages but usually occurs in children or young adults. Regular supply of insulin injections is essential for the control of glucose level in blood. The rate of *β* cell destruction varies showing fast deterioration in infants and children while the degeneration of *β* cells is slower in adults. Symptoms like ketoacidosis occur in children and young individuals while others exhibit modest fasting hyperglycemia that can change to severe hyperglycemia or ketoacidosis in response to stress or infection [22]. These patients have higher risk for developing other autoimmune disorders such as Grave's disease, vitiligo, celiac sprue, autoimmune hepatitis, myasthenia gravis, Hashimoto's thyroiditis, Addison's disease, and pernicious anemia [22]. This form of diabetes follows a hereditary pattern and is common in people of African and Asian descent [23].  Table 1 discusses the classification of the disease and the associated multiple complications.

### 2.2. Idiopathic Diabetes

A minor number of patients having type 1 diabetes, mostly of Asian and African ancestry, have no etiologies. These are prone to ketoacidosis and have permanent insulinopenia. The occurrence of ketoacidosis is in episodes and the level of insulin deficiency between episodes fluctuates. Idiopathic diabetes has genetic predisposition and an absolute need for insulin replacement therapy depends on the condition of the patient [22].

### 2.3. Noninsulin Dependent Diabetes Mellitus (NIDDM)

It is also referred to as adult onset diabetes, which accounts for 90–95% of all diabetes. Major metabolic syndromes like obesity, insulin resistance, and dyslipidaemia have led to an epidemic of type 2 diabetes [24]. The treatment of this type of diabetes is through oral hypoglycemic drugs, dietary in nature. Insulin resistance as well as loss of insulin secretion contributes to the onset of disease. Type 2 diabetes mellitus is the most common form of diabetes and is the fourth leading cause of death in developed countries with a twofold excess mortality and two- to fourfold increased risk of coronary heart disease and stroke [25].

### 2.4. Gestational Diabetes Mellitus (GDM)

It is defined as any degree of glucose intolerance resulting in hyperglycaemia of variable severity that is diagnosed during pregnancy [26]. GDM, or impaired glucose intolerance which is first diagnosed during pregnancy [27], is a major type affecting 14% women during pregnancy or 135,000 women a year in the United States and is a risk factor for type 2 diabetes in mothers [28]. The magnitude of the reported risk varies due to variations in ethnicity, selection criteria, and tests for GDM and type 2 diabetes [29]. Gestational diabetes can lead to respiratory distress syndrome, neonatal hypoglycemia, and fetal macrosomia. More infants have increased rates of birth trauma, shoulder dystocia, and cesarean delivery. Recent guidelines recommend adequate glycemic control as a strategy to decrease these maternal and fetal complications. Most women who have gestational diabetes can successfully control their blood sugar with diet and exercise, while some will require oral diabetes medication or insulin.

### 2.5. Catamenial Hyperglycaemia

Diabetic ketoacidosis (DKA) is a condition, arising due to infection, inadequate insulin or poor insulin compliance, acute pancreatitis, stroke, drugs, metabolic disturbances within the body, or negligence with the treatment [30]. The uncontrolled hyperglycaemia with DKA occurring before the menstrual cycle in females is known as catamenial diabetic ketoacidosis or catamenial hyperglycaemia. The uncontrolled hyperglycemia resulted in increased insulin requirement, up to 4 times. The condition is aggravated even after continuous insulin infusion, resulting in vomiting, and leading to significant acidosis, ketonuria, and hyperglycaemia. The strange fact was that even several tests like inflammatory markers, blood count renal function, electrocardiogram and chest radiograph, thyroid function, and urine and blood cultures were all found to be normal. The conditions leading to catamenial hyperglycaemia remain undiagnosed [31]. Hormonal changes occurring during menstrual cycle together with changes in diet and exercise levels may play a role [32]. An effective diet and exercise plans [33] including an increased insulin infusion dosage [32] will be the right medication strategy for the treatment of catamenial diabetic ketoacidosis as well as for avoiding any diabetic emergencies.

## 3. Nanotechnology and Diabetes

The interface of nanotechnology in the treatment of diabetes has introduced novel strategies for glucose measurement and insulin delivery. Researchers have demonstrated the advantages of glucose sensors and closed-loop insulin delivery approaches in facilitating the diabetes treatment to make it [34] beneficial in both type 1 and type 2 diabetes.

A nanomedical device is a microcapsule containing pores which has been a promising tool in the drug delivery approach. These pores are considerably large to allow the passage of small molecules such as oxygen, glucose, and insulin but are small enough to allow the movement of larger immune system molecules such as immunoglobulins and graft-borne virus particles. Microcapsules containing replacement islets of Langerhans cells, mostly derived from pigs, could be implanted beneath the skin of diabetes patients. This could temporarily restore the body's delicate glucose control feedback loop without the need for powerful immunosuppressants that can leave the patient at serious risk for infection [35] Table 2 describes the critical problems associated with diabetes and the role of nanomedicine in the treatment.

The nanoparticle targeted drug delivery approach has enormous benefits which include the improved bioavailability of drugs by targeting specific tissues, organs, and tumors thereby providing the highest dose of drug directly at the targeted site. One of the biggest technological challenges is the scalability of a nanoparticle. Manufacturing three-dimensional nanostructures as compared to stand-alone or two-dimensional layer-shaped nanosurfaces is a complex task since manufacturing techniques are yet to be standardized. Another apprehension is that the exposure to nanoparticles might be toxic or hazardous. Concerns about the potential ill effects of engineered nanomaterials such as carbon buckyballs and nanotubes through inhalation, ingestion, or absorption through the skin are increasing [35].

Insulin forms an essential requirement for type 1 and type 2 advanced diabetes and the traditional systems of insulin delivery included infections, painful administration, and poor compliance of patients. However, recent micro- and nanotechnologies have facilitated the insulin administration process through regulation of insulin delivery constituting pulmonary, nasal, transdermal, and closed-loop delivery [36].

## 4. Statin Therapy: A New Perspective

Statins are defined as inhibitors of 3-hydroxy-3-methylglutaryl coenzyme A and inhibit the crucial process of LDL cholesterol in liver, thereby decreasing its level in the blood besides increasing healthy blood vessel lining [37]. Since the long term effect of diabetes include the high risk of cardiovascular diseases, statins (HMG-CoA reductase inhibitor) are a main line of therapy in reducing cardiovascular risk in the patients suffering from type 2 diabetes [38, 39]. The lipid lowering agents, popularly known as statins, cause inhibition of HMG-CoA reductase specifically and reversibly. The enzyme catalyzes the conversion of HMG-CoA to mevalonic acid, the rate-limiting step in the formation of cholesterol. These compounds are highly effective in reducing cholesterol levels as compared to dietary supplements [40].

Statin therapy reduces low density lipoprotein (LDL) cholesterol to a significant level thereby greatly decreasing the chances of developing a coronary artery disease [41]. National Institute for Health and Clinical Excellence (NICE) and Scottish Intercollegiate Guidelines Network (SIGN) diabetes guidelines showed lipid lowering therapy as primary prevention (when used regularly) for patients with type 2 diabetes, aged over 40 (Grade A recommendation), as well as its consideration for patients aged over 40 with type 1 diabetes (Grade B recommendation) [41]. A recent information published at the meeting of the European association for the study of diabetes in Stockholm suggests that statin treatment is being less explored and applied in patients with type 2 diabetes among a large American group of over 100,000 subjects [42].

Statins have good efficacy and are effective in lowering cardiovascular events in people with modest levels of cholesterol and without cardiovascular disease. However, the HMG-CoA reductase inhibitors or statin therapy also has some disadvantages. The therapy has some side effects like renal dysfunction and muscle disorders from myositis to frank rhabdomyolysis and hepatic dysfunction which is rare and can be tolerated by the patient [41]. The trial conducted with 6422 patients showed that young individual and those showing absence of disease showed ineffective or poor compliance with statin therapy [38]. However, the therapy should be focused on older patients since in younger patients the poor compliance was seen. Also, the patients with high risk factors and symptoms of heart problems should be administered with statins [41]. However, reports have suggested that statins may raise the blood sugar levels moderately and lead to diabetes mellitus [37]. Despite exhibiting good toleration and less adverse effects, statins may cause side effects like myopathies and increase in levels of liver enzymes in type 2 diabetes [43].

## 5. Stem Cell Technology: A Novel Therapeutic Approach

The interest to find a possible therapeutic for diabetes has eventually explored various new scientific areas of research, with the stem cell technology being one of them. It is known that both type 1 and type 2 diabetes result from the *β* cell deficiency of the pancreatic cells, resulting in insufficient insulin secretion. The strategies should aim at either removing the defects in pancreatic *β* cell or enhancing the sensitivity of the body cells to the action of insulin. *β* cell replacement strategies offer a novel source while current strategies aiming at islet cells and pancreas transplantation are limited due to shortage of donor organs [44].

In contrast to type 1 diabetes, which is caused by autoimmune destruction of pancreatic *β* cells, type 2 diabetes results from irregularities in *β* cells function together with insulin resistance in peripheral organs [45]. Mesenchymal stem cell (MSC) therapy has emerged as a promising therapy in the treatment of type 1 diabetes due to its immunosuppressive nature. MSCs have been found to display immunomodulatory effects both in* in vitro* and* in vivo* conditions due to direct contact and production of soluble markers [46–49]. MSCs have the potential to differentiate into a number of mesenchymal cell lineages. The hematopoietic stem cells are the multipotent stem cells that can give rise to all the cell type in blood and also possess immunomodulatory effect. Hence, the transplantation of hematopoietic stem cell has proved to be a promising therapeutic, resulting in improvement in *β* cell function in newly diagnosed type 1 diabetic patients [50]. Further studies have demonstrated that the induced pluripotent stem (iPS) cells can be generated from type 1 diabetic patients by reprogramming their adult fibroblasts with three transcription factors (OCT4, SOX2, and KLF4). The cells known as diabetes induced pluripotent stem cells; (DiPS) are pluripotent and have the ability to differentiate into insulin producing cells. This is beneficial in type 1 disease modeling and cell replacement therapies [51].

Some studies have shown that bone marrow derived MSCs have the ability to differentiate into insulin producing cells both* in vitro* and* in vivo* [52–54]. The significance of human embryonic stem cells (ESCs) in the treatment of diabetes has attracted great attention due to their pluripotent nature and large scale production of different cell lineages in cultures. The research has various limitations since there is absence of reliable methods for generating specific cell types, immunological rejection of the transplanted cells, and difficulty in purification of specific lineages [55]. Further concerns include the uncontrolled proliferation of the transplanted embryonic stem cells into a specific type, once they are transplanted [56]. Still, despite of its manifold limitations both scientific and ethical, the application of stem cell technology holds immense prospects in treatment of diabetes.

## 6. Gene Therapy in Diabetes

The series of experiments leading to cloning and expression of insulin in the cultures cells in the 1970s was a tremendous revolution in the field of medicine and application of gene therapy in the treatment of diabetes was suggested as a possible cure. Regulating the sugar levels is the most important aspect in the treatment which also reduces the complications associated with the disease. Somatic gene therapy involving the somatic cells of the body includes two methods of gene delivery. The first one known as* ex vivo* gene therapy is described as the one in which the tissues are removed from the body; the therapeutic gene is inserted* in vitro* and then reimplanted back in the body while the* in vivo* therapy involves the insertion of gene therapy vectors directly to the patients by subcutaneous, intravenous, or intrabronchial routes, or by local injection [57]. The application of* ex vivo* therapy aims at the generation of cells which possess the properties of *β* cells, for example, insulin producing cells [58]. This therapy has also been used to generate *β* cells for transplantation. However, the concern lies in the aspect of surgically removing the tissue from the patient and reimplantation of the genetically modified tissues back into the body of the patients [57]. Furthermore, type 1 diabetes results from autoimmune destruction of insulin synthesizing pancreatic *β* cells and islet transplantation has been explored as a possible solution for the treatment. The invention of insulin gene therapy substitutes *β* cell function by generating insulin secretory non-*β* cells, not vulnerable to autoimmune reactions, offering a prospective therapeutic approach for type 1 diabetes [59].

The* in vivo* gene therapy is the method of choice as a therapeutic strategy because it is simpler and the vector containing the desired gene is directly inserted into the patient, but the development of safe (not toxic to host) and effective vectors remains as a challenging task for gene therapist. Presently, the strategies for* in vivo* therapy involve three methods: genetic transfer of glucose lowering genes which are noninsulin in nature. Presently, the strategies for* in vivo* therapy include genetic transfer of glucose lowering genes which are non-insulin in nature and application of blood sugar lowering genes: an enhancer of glucose utilization by liver or skeletal muscles and an inhibitor of glucose production by the liver [57]. For example, glucokinase as a transgene is found to have glucose lowering effect in the liver [60]. It was a possibility that the gene* Gck* enhances glucose utilization by the body [61]. The genetic transfer of glucokinase had been used as an adjuvant therapy in the treatment of diabetes [62]. In another strategy which was carried out to regulate the glucose production in liver, a gene known as “protein targeting to glycogen” (PTG) was used to convert glucose to glycogen [63, 64]. The PTG protein belongs to the family of glycogen targeting subunits of protein phosphatase-1 which regulated the metabolism of glycogen. Experiments performed in rats have indicated that adenoviral mediated PTG transfer stimulates glycogen synthesis in the liver and decreases blood glucose levels in rats. This has been considered as a therapeutic approach for diabetes [63].

Other areas of genetic engineering include transfer of genes which show response to glucose and the use of gene therapy to induce *β* cells production in the liver [57]. The glucose responsive genes that have been manipulated to enhance conversion of proinsulin to insulin and those which after modification exhibit expression show responses to blood glucose level [65, 66]. The liver cells do not produce hormones which convert proinsulin to insulin; therefore, new proteolytic cleavage sites have been incorporated into the proinsulin molecule, recognized by a protease, furin that is present in many tissue systems, including liver [67–69]. The insulin gene can be modified to encode insulin which has single-chain [70] having 20–40% activity of normal mature insulin [71].

Research has also been carried out to induce the synthesis of *β* cells formation in the liver. Kojima et al. reported that it is possible to induce the formation of *β* cells by the endocrine cells by delivering islets specific transcription factors [72, 73]. The regulation of insulin production and its control remains as a difficult task since the knowledge about insulin metabolism is much less [74]. The strategy aiming at induced *β* cells neogenesis seems to be a promising approach as a therapeutic for diabetes, since it can offer a solution for the autoimmunity in type 1 diabetes [57].

## 7. Medical Nutrition Therapy

Medical nutrition therapy in prevention and management of diabetes puts forth numerous advances in clinical research, aiming to use nutrition therapy for the treatment of disorders and illnesses. American Diabetes Association in 1994 coined the term “medical nutrition therapy” constituting 2 phases, namely, adjudging the nutritional requirement of a person and treatment through counseling and nutrition therapy, respectively [75]. The objectives of nutritional therapy in diabetes is to regulate optimal level of lipids in blood, ideal body weight, and blood glucose level in normal range. Nutrition therapy as a therapy for diabetes depends on certain factors such as patient's age-based nutritive requirements and food preferences as well as other medical conditions together with an exercise regime and recommended nutritional requirement depending upon the patient's abilities and health conditions [76, 77]. Calorie requirement to maintain ideal body weight for moderately active individual is 30–35 kcal/kg/day; for obese people it is 20–30 kcal/kg/day. It is estimated that gradual weight loss of 1 lb per week should occur, if the calorie intake is reduced by 500 calories/day [76, 77]. According to recent recommendations, the percent of carbohydrate intake is based on the patient's intake of protein and fat. Low carbohydrate/high protein diet is popular and may be associated with initial weight loss and improved glycemic control but is difficult to maintain for longer time periods. Protein intake is maintained at 10–20% of all calories; total fat intake should be restricted to <30% of total calories; high fibre diet (20–35 g/day of soluble and insoluble fiber), sodium restriction to 2400–3000 mg/day, alcohol intake (≤2 drinks/day in men, ≤1 drink/day in women), and multivitamins should be taken in the diet [76, 77].

## 8. Natural Products and Diabetes

Literature has suggested the utilization of herbal medications for the treatment of insulin dependent and noninsulin dependent diabetes since time immemorial. Plants possessing antidiabetic properties may be suitable as adjunct to the existing therapies or as a prospective source of new hypoglycemic compounds. Since time immemorial, naturopathic therapies have been applied for a number of health ailments and continue to gain popularity in the present arena as well. Ancient literature revealed that diabetes was a known disease since Brahmic period and finds a mention in Ayurvedic literature, Sushruta samhita written in fourth and fifth centuries BC [78]. Two forms of diabetes were described: one genetic in nature and the other due to dietary indiscretion [78]. Herbal medicines are becoming immensely popular among the masses for being cost effective and with relatively few side effects. Although plant based medicines have been used traditionally in treating diseases throughout the world, the mechanism of most of the herbs is still to be defined and standardized [79]. Many new bioactive drugs isolated from plants having hypoglycaemic effects demonstrate antidiabetic activity equal to and sometimes even more potent than known oral hypoglycaemic agents such as daonil, tolbutamide, and chlorpropamide. However, many other active agents obtained from plants have not been well characterized [80]. Grover et al. [81] postulated that plants possessing antidiabetic activities are of significant interest for ethnobotanical community as they are recognized to contain valuable medicinal properties in different parts and a number of them have shown varying degree of hypoglycemic and antihyperglycemic activity. The bioactive constituents found in many plant species are isolated for direct use as drugs lead compounds, or pharmacological agents. These traditional approaches might offer a natural key to unlock diabetic complications [82]. The chemical structures of a phytomolecule play a critical role in its antidiabetic activity. Several plant species being a major source of terpenoids, flavonoids, phenolics, coumarins, and other bioactive constituents have shown reduction in blood glucose levels as demonstrated by Jung et al. [83]. Several plants like* Allium sativum* Linn. (Liliaceae),* Gymnema sylvestre *(Retz.) Schult (Asclepiadaceae),* Murraya koenigii* (L.) Spreng. (Rutaceae),* Allium cepa* (Liliaceae),* Withania somnifera* Dunal (Solanaceae), and* Ferula foetida* Linn. (Umbelliferae) have been found to possess antidiabetic properties when assessed in experimental models of diabetes. The antidiabetic properties of* G. sylvestre *had been discussed in detail [84, 85] owing to its significance in diabetes treatment and management.

## 9. Future Perspectives

Diabetes has remained as one of the most challenging health problems in the 21st century accounting for a global presence. Diabetes is a serious public health problem, but the good news is that important advances are being made in prevention, detection, and treatment of diabetes. For the management of type 1 diabetes, patients require insulin administration 3-4 times a day throughout their lives and their blood sugar levels should be regularly monitored to avoid complications like retinopathy and risks of cardiovascular diseases. It has been estimated that around 1300 patients with type 1 diabetes receive whole organ (pancreas) transplant and do not require insulin infusion but the demand for organs transplantation is higher than supply. Another risk factor is the rejection of transplanted organ; therefore, patient is given strong immunosuppressive drugs which can lead to other serious diseases [86].

For the management of type 2 diabetes, a well monitored glycemic control is required. The need to control the progressive deterioration of *β* cell function is essential since it can lead to a loss of glycemic control. Conventional drugs and insulin are effective but cannot repair the associated metabolic and glucoregulatory dysfunctions. The menace of diabetes is increasing day by day and aggressive and targeted combinational therapy is the need of the hour particularly incretin based therapy and peptide analogs. This may restore and preserve *β* cell function and halt the progression of type 2 diabetes [87]. In the present era, the effectiveness and the success of the new drug will depend on its ability to treat/relieve one or more of the metabolic disturbances whether increased production of insulin or enhancement in glucose uptake and utilization by the peripheral tissues particularly skeletal muscle. Besides new generations of therapeutics, several other classes have also been reported as alternative strategies alone or in combinations to provide an effective treatment for diabetes.

The prospects of leptin therapy are one of the emerging trends in the treatment of diabetes. It is a hormone secreted by adipocytes, which acts on the neurons within the central nervous system. The multiple actions of this hormone include control of excessive increase in weight, by suppressing the intake of food and increasing the expenditure of energy [88]. Leptins also regulate glucose homeostasis through the activation of leptin receptors (LEPRs) [89–92]. It has been shown that the central nervous system regulates the sugar lowering effect of leptins; it was assumed that the antidiabetic action of leptins could have been influenced by neurons in the brain with reference to type 1 diabetes. Leptin therapy improves insulin-deficient type 1 diabetes by CNS-dependent mechanisms in mice [93].

Another area of drug research includes designing and use of mucoadhesive microcapsules of various drugs like glipizide to achieve controlled release of the drug and its effective targeting. Mucoadhesion has been a novel approach in drug delivery designing because it causes the slow release of the drug at the action or absorption site thereby enhancing the interaction of the drug with the underlying tissue forms, enhancing the bioavailability of the drugs [94]. There is no end to the drug delivery approaches which have been followed as a possible cure for diabetes. The transdermal insulin administration approach (which has been developed as a consequence of painful and complicated insulin therapy) maintains constant levels of insulin without the deposits of insulin in the skin frequent with subcutaneous insulin injections (http://www.ondrugdelivery.com/, 2006). A research by Odegaard and colleagues revealed that activated macrophages display a beneficial role in the regulation of nutritional homeostasis and suggests that polarization of the macrophages towards the alternative state might be a useful possibility in the treatment of type 2 diabetes [95].

Great strides have been made clinically in the prevention, development, and treatment of the disease but no therapeutic method have been completely successful till date. With new technologies revolutionizing the treatment possibilities, the search for an effective medication is not far ahead. The extensive research leading to the discovery of the pathway genes contributing to the development of the disease and the sequencing of complete genomes have revolutionized the diabetes research. The development of the techniques like the PCRs, DNA microarray, and gene knockouts with silencing has opened up a new area in the identification of the defective genes/mutations in the genome of the organism. The increasing prevalence of diabetes globally is creating a financial burden on the economy of the respective country. Unlike some other diseases, treatment exists for diabetes, and if managed correctly, it is very effective in reducing complications such as heart attacks, amputations, blindness, and kidney failure. With the ongoing research, a right therapeutic for the treatment of diabetes is not unachievable.

## Figures and Tables

**Table 1:** Representation of the multiple primary and secondary forms of diabetes, the defective metabolism, and the adverse effects on the body organs.

Classification of diabetes	Effects on the body	Symptoms
Primary diabetes		
Insulin dependent diabetes mellitus	Destruction of *β* cells	Deficiency of insulin
Non insulin dependent diabetes mellitus	Insulin resistance	Loss of insulin secretion

Secondary diabetes/symptoms due to primary diabetes		
Hormonal imbalance	Acromegaly Pheochromocytoma	
Pancreatic dysfunction	Pancreatitis Pancreatectomy Cushing's syndrome Glucagonoma	
Drugs or chemical induced reactions, for example, anticancer agents Thiazide Psychoactive agents like glucocorticoids, streptozotocin, or diazoxide	Drug induced reactions	Hypersensitivity reactions
Insulin receptor abnormalities	Genetic syndromes Hyperlipidemia Muscular dystrophy	
Malnutrition	Hyperglycemia	Enhanced sugar levels
Glycosuria	Glycosuria Loss of weight	Excessive secretion of sugar in urine
Ketonuria	Ketosis and elimination in urine Dehydration	
Lipemia	—	Increased levels of lipid, fatty acids, and cholesterol in blood
Acidosis	—	Lowering of pH of blood
Cataract and lesions of blood vessels (atheromatous, and artherosclerotic)		

**Table 2:** Description of some problems associated with diabetes and possible nanomedicine solutions.

Measurement problems	Nanometrology solutions
Continuous blood glucose monitoring Stable implanted enzyme electrodes Noninvasive monitoring	Biocompatible nanofilms “Smart tattoo” of glucose Nanosensors

Improved diagnosis Targeted molecular imaging Understanding mechanisms	NIR QDs, gold nanoparticles Single-molecule detection

Therapy problems	Nanotherapeutic solutions

Improved insulin delivery Islet cell transplantation Oral insulin Closed-loop insulin delivery	Islet nanoencapsulation Insulin nanoparticles Artificial nanopancreas
